# Sub-Micron Particle Based Structures as Reconfigurable Photonic Devices Controllable by External Photonic and Magnetic Fields

**DOI:** 10.3390/s110302740

**Published:** 2011-03-02

**Authors:** Asaf Shahmoon, Amihai Meiri, Zeev Zalevsky

**Affiliations:** School of Engineering, Bar-Ilan University, Ramat Gan 52900, Israel; E-Mails: meiria1@gmail.com (A.M.); zalevsz@eng.biu.ac.il (Z.Z.)

**Keywords:** optical resonant, plasmon resonance, nanoplasmonic sensor, microsensor chip, micro arrays

## Abstract

In this paper we present the configurations of two nanometer scale structures—one of them optically controllable and the second one magnetically controllable. The first involves an array of nanoparticles that are made up of two layers (*i.e*., Au on top of a Si layer). The device may exhibits a wide range of plasmonic resonance according to external photonic radiation. The second type of device involves the usage of sub micron superparamagnetic particles located on a suitable structuring grid, that according to the angle of the external magnetic field allows control of the length of the structuring grid and therefore control the diffraction order of each wavelength.

## 1. Introduction

Sub-micron metallic as well as superparamagnetic particles that are incorporated into special structures, may exhibit various interesting properties that may be used for sensing as well as for information manipulation when interacted with photonic radiation, thus being applicable as part of reconfigurable photonic devices [1]. Nanophotonic devices that incorporate the usage of metallic nanoparticles have a large variety of multidisciplinary functionality and applicability in fields such as biology, photonics and engineering. The main advantages of these photonic devices are their small size, lower power consumption, low cost and high modulation rate.

In the past few years there has been great interest in characterizing the optical properties of non-spherical nanoparticles, such as triangular [2,3], nano disk [4,5] and especially nanorod shapes [6,7] made from different materials such as silver, Au and semiconducting materials. The plasmonic resonance of nanoparticles strongly depends on their size, geometry, internal morphology and the dielectric constant of the surrounding medium. The discrete dipole approximation (DDA) method [8] can calculate the extinction cross section of nanoparticles with arbitrary shape and for complex refractive index and has been used in order to compute the extinction cross section of the proposed devices.

In this paper we focus on two types of device configurations. The first involves metallic nanoparticles or nanorods incorporated into a semiconducting structured matrix that exhibits strong plasmonic resonance which depends on the power of the external photonic radiation that is being absorbed by the matrix. This spectral shifting of the resonance allows the realization of a (photonic) intensity controllable spectral reflecting structure capable of reflecting another, low power, incident photonic radiation. The second type of structures that is described in this paper involves usage of sub-micron superparamagnetic particles embedded into a suitable structuring grid where, this time, the intensity of an external magnetic field controls the reflectance properties of an incident photonic radiation. Both devices may be used, among other applications, as sensing devices where in the case of the first device, the sensing is related to the incoming photonic radiation and in the second device, the sensing operation is related to the intensity of the external magnetic field. The remainder of the paper is organized as follows: in Section 2 we present the operation principles as well as a numerical analysis of the plasmonic resonance device. In Section 3 we describe the superparamagnetic device as well as its preliminary experimental validation. The paper is concluded in Section 4.

## 2. Plasmonic Resonance Device

### 2.1. Operation Principle

**Figure 1.**
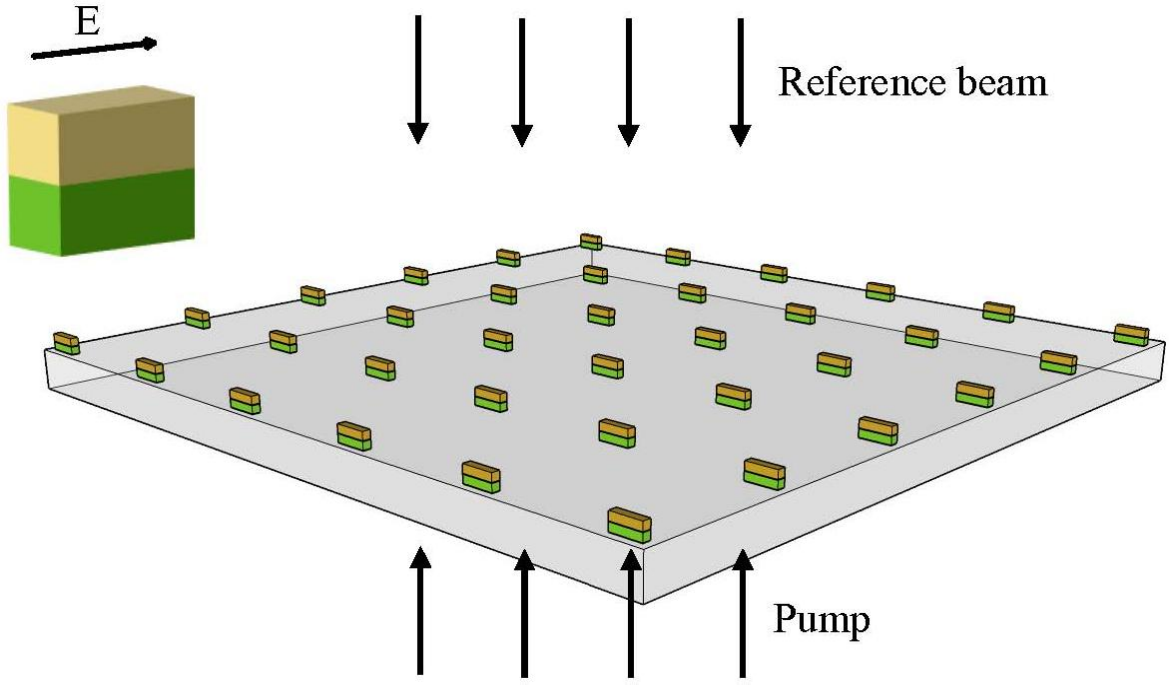
Schematic sketch of the system.

Figure 1 shows a conceptual sketch of the device. One may see the array of the nanoparticles that are made up of two layers (Au on top of a Si) that are being illuminated by two light beams. On the upper left corner we present the polarization of the electric field relative to a single nanoparticle. Note that we refer to longitudinal oscillation only where the E-field is parallel to the long axis of the nanoparticle. The transverse case does not depend on the aspect ratio of the nanoparticle and therefore no tunable effect can be obtained. We note that the device presented in Figure 1 uses an array of nanoparticles in order to increase the accumulated effect.

The structure is continuously illuminated by a uniformly distributed beam at the wavelength’s range of interest. When the pump illumination is absent we have light excitation at a wavelength that corresponds to the geometry of the device [9]. By using a high intensity pump illumination which is absorbed by the silicon nanorod at the bottom, the free charge carrier density is changed and thus the refractive index of the device. When the carrier density inside the silicon nanorod is high enough we essentially obtain a metallic like nanorod attached to the gold nanoparticle which results in a different aspect ratio. Using this mechanism we can control the aspect ratio of the structure by means of external illumination, thus changing the wavelength of the peak power excitation.

The mechanism of change in the refractive index in silicon as a function of the carrier concentration is known as the plasma dispersion effect. This change is given by (1) [10]:

(1) $$\Delta n=\frac{-e^{2}\lambda_{0}^{2}}{8\pi^{2}c^{2}{ɛ}_{0}n}\left(\frac{{\Delta N}_{e}}{m_{e}^{*}}+\frac{{\Delta N}_{h}}{m_{h}^{*}}\right)$$

For the real part of the refractive index, the change in the absorption coefficient equals:

(2) $$\Delta \alpha =\frac{e^{3}\lambda_{0}^{2}}{4\pi^{2}c^{3}{ɛ}_{0}n}\left(\frac{{\Delta N}_{e}}{\mu_{e}{\left(m_{e}^{*}\right)}^{2}}+\frac{{\Delta N}_{h}}{\mu_{h}{\left(m_{h}^{*}\right)}^{2}}\right)$$

where *e* is the electron charge, λ_0_ is wavelength of light in free space, *c* is the speed of light, *ɛ_0_* is the vacuum permittivity, *n* is the refractive index, Δ*N_e_* and Δ*N_h_* are the change in the carrier concentration of the electrons and holes, respectively. *μ_e_* and *μ_h_* are the mobility of the electron and holes. m^*^_e_ and m^*^_h_ are the effective electron and hole masses, respectively. The change of the free carrier concentration is:

(3) $${\Delta N}_{e}={\Delta N}_{h}=\frac{\eta \times P}{h\times \nu }$$

where *η* is the quantum efficiency, *P* is the intensity of the pump illumination and *hυ* is the energy of each photon (*h* is Plank’s constant and *υ* is the optical frequency of the photon). From equations (1) and (2) we can see that the pump illumination causes the real part of the refractive index to decrease, while increasing the imaginary part of the refractive index, thus obtaining values close to that of a metal.

### 2.2. Numerical Analysis

**Figure 2.**
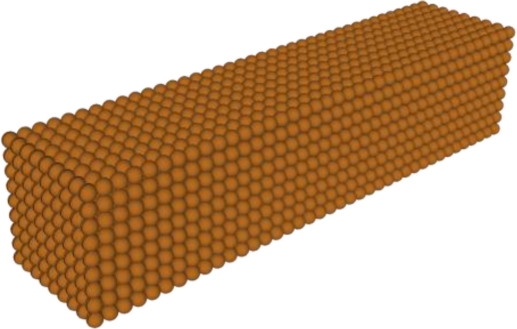
Array of dipoles constructing the target geometry, a single nanoparticle.

The Discrete Dipole Approximation (DDA) is a flexible method for computing the scattering and absorption by particles with arbitrary geometry. In this method, the Maxwell’s equations are calculated by dividing the geometry of a single nanoparticle into a target array of N dipoles (j = 1…N) as presented in Figure 2. For each dipole the solution for the oscillating dipole moment can be found and the scattering and absorption cross sections are then calculated.

The electric field at location *r_j_* can be written as the sum of incident electric field and the effect of the other N-1 dipoles:

(4) $$E_{j}=E_{\mathrm{inc},j}-\sum_{k\neq j}{\text{A}}_{\mathrm{jk}}\times {\text{P}}_{k}$$

where *E_inc,j_* is the incident electric field, *A_jk_* is an interaction matrix between *j* and *k* dipoles and *P_k_* is the dipole moment at the *k*'th element. A is a *N* × *N* interaction matrix whose elements are 3 × 3 tensors which can be defined by:

(5) $$\begin{array}{l} A_{\mathrm{jk}}=\frac{\text{exp}({\mathrm{iKr}}_{\mathrm{jk}})}{r_{jk}}\times \left[k^{2}\left({\hat{r}}_{\mathrm{jk}}{\hat{r}}_{\mathrm{jk}}-1_{3}\right)+\frac{{\mathrm{iKr}}_{\mathrm{jk}}-1}{r_{\mathrm{jk}}^{2}}\left(3{\hat{r}}_{\mathrm{jk}}{\hat{r}}_{\mathrm{jk}}-1_{3}\right)\right],\;j\neq k\\ {\text{A}}_{\mathrm{jj}}=\alpha^{-1} \end{array}$$

where *K* is the wave vector, *r_jk_* is the distance between *j* and *k* dipoles, *r̂*_*jk*_ is a unit vector and *α* is the polarizability which is geometry dependent and takes into account the dielectric constant of the nanoparticles and the surrounding medium. Using these relations, a system of equations can be solved in order to find the dipole moments:

(6) $$A\times P=E_{\text{inc},j}$$

The extinction and absorption cross sections can be evaluated by:

(7) $$C_{\mathrm{ext}}=\frac{4\pi K}{{\left|E_{0}\right|}^{2}}\sum_{j=1}^{N}\text{Im}\left(E_{\mathrm{inc},j}^{*}\times P_{j}\right)$$

and:

(8) $$C_{\mathrm{abs}}=\frac{4\pi K}{{\left|E_{0}\right|}^{2}}\sum_{j=1}^{N}\left\{\text{Im}\left({\text{P}}_{j}\times {\left(\alpha^{-1}\right)}^{*}\times P_{j}^{*}\right)-\frac{2}{3}K^{3}{\left|P_{j}\right|}^{2}\right\}$$

where the scattering cross section is *C*_*sca*_ = *C*_*ext*_ − *C*_*abs*_ and * denotes the complex conjugate. The numerical analysis of the device was carried out using the freely available DDSCAT 7.0 [11] software which uses DDA method to calculate the scattering and absorption of nanoparticles with arbitrary shape and complex refractive index.

The resonance wavelength excitation was tested at the range of 300 nm–700 nm, while the wavelength step was chosen to be 2 nm at the critical area (at the peak region) and 10 nm outside the peak region. The distance between dipoles was chosen to be 1 nm, in order to obtain accurate results while not increasing the computational cost significantly (the inter dipole distance affects the accuracy of the numerical solution while a denser inter dipole matrix will improve the accuracy of the solution, while increasing the computational cost). The polarization of the incident light was parallel to the long axis of the nanoparticle. The propagation direction was as indicated in Figure 1. The cross section of each nanoparticle was 10 nm × 10 nm while a comparison between nanoparticles with lengths of 40 nm and 50 nm (*i.e*., different aspect ratio) was performed. For the 40 nm nanoparticles we also investigated the behavior of a single elongated Si nanoparticle which has the overall size equal to the original size of the Si and Au nanoparticles when placed one on top the other.

**Figure 3.**
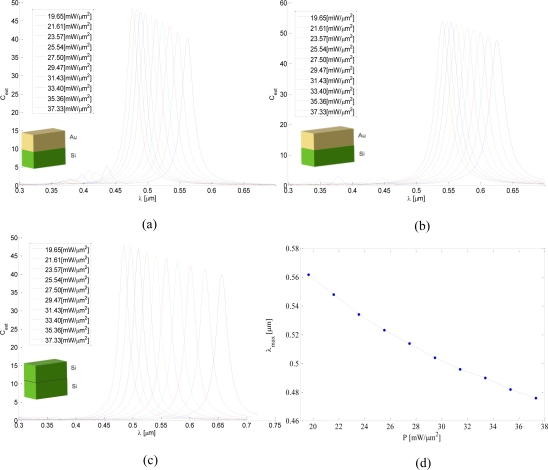
Simulation Results: **(a)** and **(b)** extinction cross section calculated for 40 nm long and 50 nm Au-Si structures, respectively; **(c)** Extinction cross section calculated for a Si nanoparticle; **(d)** Peak wavelength as a function of the pump intensity for a 40 nm long Au-Si structure.

The simulation results can be seen in Figure 3. Figure 3(a) shows the absorption cross section calculated for a 40 nm long structure with different pump illumination intensities. One may see that as the intensity of pump illumination increases the absorption peak and its wavelength shift from 562 nm with a pump of 19.65 mW/μm^2^ towards the blue wavelength of 476 nm with a pump of 37.33 mW/μm^2^. While the 50 nm particle exhibits the same behavior, but the entire image is red shifted, see Figure 3(b). These results can be explained by the fact that the higher the aspect ratio is, the higher the peak wavelength is. Illuminating the silicon with the pump increases its free carrier concentration towards that of the gold nanoparticle to a point where it becomes a single and effectively thicker gold nanoparticle and thus has a smaller aspect ratio.

The results for the Si single nanoparticle, shown in Figure 3(c), indicate that the single silicon particle can have a broader range of peak absorption wavelengths (range of 484 nm to 656 nm in comparison to a range of 476 nm to 562 nm for the case of gold-silicon structure). However, looking at the width of the peaks we can see that silicon has a full width half maximum (FWHM) which is 10% larger than that of the silicon-gold structure. Note, that for high pump illumination the results for a single silicon nanoparticle and a Au-Si structure show the same location for the peak, which indicates that for that intensity silicon becomes as conductive as gold.

The dependence of the peak location on the pump illumination intensity is depicted in Figure 3(d). According to the results, the dependency between the peak wavelength and the pump intensity is linear, thus we used a linear fit to obtain the equation of the peak wavelength *λ*_max_[*μm*] = −4.777×10^−3^*P*+649×10^−3^ for the 40 nm long nanoparticle. Since the linear relation between the absorption peak wavelength and the aspect ratio is also known [12], we can design the device to have the exact desirable peak wavelengths.

### 2.3 Experimental Results

**Figure 4.**
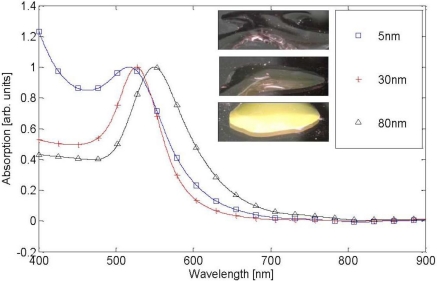
Measured absorption spectra of gold nanospheres.

We examined the absorption of Au nanospheres in water using Shimadzu UV-1650pc spectra-photometer. The results can be seen in Figure 4.

The purpose of this experiment was to show that indeed as previously claimed, changing the geometrical properties or the conductivity of nanoparticles (which can be controlled by external illumination) affect their spectral behavior. The size of the nanospheres was 5, 30 and 80 nm and the absorption spectra resulted peaks were at wavelengths of 517, 526 and 549 nm, respectively.

We can see that by enlarging the nanospheres we can obtain a red shift of the spectra (the colored images as captured by the spectrophotometer are seen in the right upper corner of the figure). We can also see that while the 5 nm particles have no distinct peak, the 80 nm particles have a distinct peak which is wider in its area than that of obtained for the 30 nm particles. The experimental results presented in Figure 4 fit well with the previously presented numerical/mathematical modeling.

### 2.4. Devices and Fabrication Process

**Figure 5.**
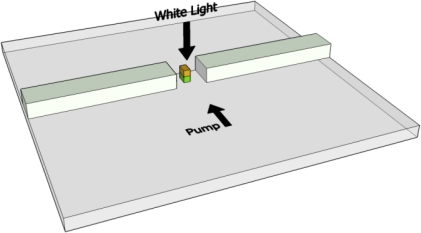
All-optical modulator based on plasmon resonance effect.

The demonstrated concept can be used for the realization of sensing as well as reconfigurable all-optical devices. In case of a sensor application the output of the device is a photonic output which provides photonic readout according to the external intensity of the pump illumination that is being illuminated over the device shown in Figure 1. In addition, a configuration such as the one shown in Figure 1 can be used as a multiplexer where the pump illumination controls the output of the device by selecting the proper input wavelength channel. Furthermore, the device can be used as an optical switch as well as a tunable filter which is polarization dependent in optical communications links. Figure 5 depicts an example of a nanoparticle based all-optical modulator. Here we have a waveguide with a nanoparticle inside an air gap. The particle is illuminated from above with a white light. By designing the waveguide in such a way that it supports only guided modes for wavelengths that are smaller than the zero pump peak wavelength of the particle, we obtain radiative modes when the pump illumination is absent and therefore the optical power is not guided towards the output. When the pump is present we obtain guided modes and then the power is propagated towards the output.

**Figure 6.**
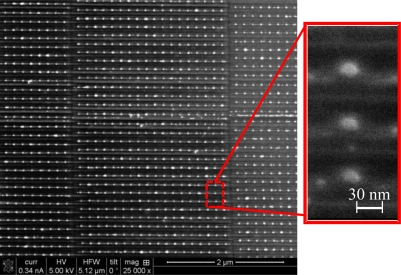
SEM image of the fabricated device.

Figure 6 shows a top view Scanning Electron Microscope (SEM) image of the device reported in Figure 1. The fabrication itself included several stages. First we used the Bestec system to evaporate 10 nm of silicon on top of a silica wafer. Right after, 10 nm of Au was evaporated again on top of the silicon layer. The next stage was to etch the surface, using the Focused Ion Beam (FIB) in such a way that an array of nanorods that are made out of two layers (*i.e*., Au on top of a silicon layer) was generated.

## 3. Superparamagnetic Device

The second type of structures described in this paper involve the usage of sub-micron super-paramagnetic particles [13] embedded into a suitable structuring grid where, this time, the intensity of an external magnetic field is the one controlling the reflectance properties of an incident photonic radiation. In the experiments we used superparamagnetic magnetic microparticles in an aqueous suspension (SiMAG-Silanol) and having a diameter of 0.5 μm. The magnetic micro particles were manufactured by Chemicell, Berlin, Germany (product number 1101–5).

Superparamagnetism is a form of magnetism, which appears in small ferromagnetic or ferrimagnetic nanoparticles. In small enough nanoparticles, magnetization can randomly flip direction under the influence of temperature [14]. When an external magnetic field is applied to superparamagnetic nanoparticles, they tend to align along the magnetic field, leading to a net magnetization. The magnetic susceptibility of the nanoparticle χ is defined as:

(9) $$\chi =\frac{N\;\mu_{0}\;\mu^{2}}{3k_{B}T}$$

where μ_0_ is the vacuum permeability, N the number of identical nanoparticles with randomly oriented easy axis, μ is the magnetic moment carried by one nanoparticle, k_B_ the Boltzmann constant and T is the temperature.

**Figure 7.**
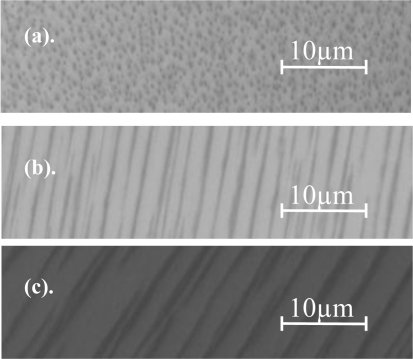
Superparamagnetic micro particles. **(a)** Microparticles in absence of external magnetic field; **(b)** and **(c)** Structure of the microparticles under the influence of increasing external magnetic field.

Figure 7 presents the superparamagnetic particles. Figure 7(a) shows the initial state of the magnetic microparticles in the absence of external magnetic field, where a monodisperse particle distribution is being shown, while in Figures 7(b) and 7(c) we present the change of their spatial periodicity as function of increased external magnetic field [the field in Figure 7(c) is eight times larger than the one of Figure 7(b)], which directly affects the wavelength being reflected from the structure when illuminated by photonic illumination. Once again the proposed device can be used as a sensing device in which the reflected wavelength is related to the intensity of the applied external magnetic field. Moreover, the aforementioned configuration can also be used as photonic reconfigurable filters or Wavelength Division Multiplexing (WDM) multiplexers that are being controlled and reconfigured by external magnetic field.

**Figure 8.**
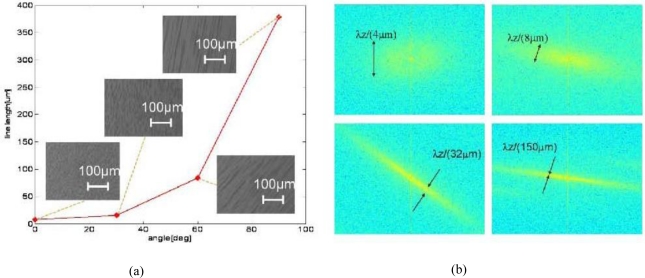
Superparamagnetic particle angle dependent. **(a)** Plot of the aligned line array as a function of the magnet angle; **(b)** Spectrum of the aligned lines corresponding to the angle of the magnet.

In addition, by changing the angle of the magnetic field we are able to control the length of the aligned lines. The experimental results are presented in Figure 8. Figure 8(a) presents a plot of the physical length of the aligned lines array formed by the micro particles as a function of the angle of the magnet. Where zero angle correspond to a magnet that one of its poles is located normal to the optical axis of the light coming from the microscope.

Here one may see that the lines become elongated as the angle increases. In Figure 8(b) we present the spectrum of experimentally obtained lines corresponding to the angle of the magnet. The spectrum was calculated using the FFT function via the Matlab software. As long as the lines become larger the spectral peaks become narrower. This effect strongly depends on the wavelength of light and therefore the device can be used for different spectral filtering applications.

## 4. Conclusions

In this paper we have presented two types of devices that are controlled by external means. The first type of device, which involves an array of nanoparticles that are made up of two layers, has the ability to control the plasmon resonance wavelength according to the external photonic radiation. The second type of device, which involves superparamagnetic particles, has spectral (wavelength) selectivity obtained according to the externally applied magnetic field.

In addition, both types of sub-micron particle-based devices can be used for sensing while for the metallic particles structure it is the sensing of photonic radiation and for the case of the superparamagnetic particles based device it is the sensing of external magnetic field. The anticipated performance of the proposed devices was investigated.
